# Neutropenia in patients with adenosine deaminase deficiency

**DOI:** 10.1186/1710-1492-10-S1-A41

**Published:** 2014-03-03

**Authors:** Vy HD Kim, Chaim R Roifman, Eyal Grunebaum

**Affiliations:** 1 Division of Clinical Immunology and Allergy, Department of Paediatrics, Hospital for Sick Children and University of Toronto, Toronto, Ontario, Canada

## Background

Adenosine deaminase (ADA) deficiency is a disorder where the accumulation of purine metabolites, which are particularly toxic to lymphocytes, can lead to severe, life threatening infections. In addition, ADA deficiency also affects other tissues. Few reports describe the presence of neutropenia in these patients, primarily in older patients after hematopoietic stem cell transplantation (HSCT) or gene therapy. We hypothesized that abnormal purine metabolism could also affect granulopoiesis.

## Objective

The objective of the study was to assess the frequency and nature of neutropenia in patients with ADA deficiency in the first 180 days of life.

## Methods

This retrospective study analyzed all patients who were diagnosed with ADA deficiency at the Hospital for Sick Children between 1984 and 2012 and had at least one documented complete blood count with differential in the first 180 days of life.

A diagnosis of ADA deficiency was made when erythrocytes ADA enzyme activity was less than 1-2% of control and/or demonstration of mutations in the ADA gene.

Neutropenia was defined as the absolute neutrophil count of less than 6.0 x 10^9^/L for ages ≤ 6 days, less than 1.5 x 10^9^/L for ages 7-13 days, less than 1.0 x 10^9^/L for ages 14-89 days and less than 1.5 x 10^9^/L for ages ≥ 90 days. Patients were excluded if neutropenia was first documented after chemotherapy for HSCT.

## Results

Thirteen patients with ADA deficiency were included in the study. Nine of the 13 patients had neutropenia that was first documented within the first 180 days of life (median age of first neutropenia 70 days, range 1-176 days). In 5 patients, the neutropenia was not present on the initial blood count but developed over time. The lowest neutrophil counts ranged from 0.11-1.08 x 10^9^/L (median 0.5 x 10^9^/L). The neutropenia developed in 7 patients prior to the onset of cotrimoxazole or other medications that commonly have myelosuppressive effects. The neutropenia was not associated with infections commonly causing neutropenia or with autoimmune manifestations. Bone marrow examinations in 2 patients with neutropenia were reported as normal. The neutropenia improved spontaneously in 3 patients, while in 4 additional patients it resolved after initiation of PEG-ADA replacement therapy or HSCT.

## Conclusions

Neutropenia occurs commonly in patients with ADA deficiency. Further studies are required to determine the pathogenesis of the neutropenia in ADA deficiency.

